# P-1001. Utilizing Perspectives of Prenatal Providers to Explain the Rise of Congenital Syphilis in Chicago, USA: A Mixed-Methods Study

**DOI:** 10.1093/ofid/ofae631.1191

**Published:** 2025-01-29

**Authors:** Nikki Kasal, John Flores, Caroline Montag, Yeo Won Ahn, Jackson Montgomery, Daniela Zimmer, Alicia Dawdani, Ellen Almirol, Jessica Ridgway, John Schneider

**Affiliations:** University of Chicago Pritzker School of Medicine, Chicago, Illinois; University of Chicago Hospital, Chicago, Illinois; University of Chicago Pritzker School of Medicine, Chicago, Illinois; The University of Chicago, Chicago, Illinois; University of Chicago, Chicago, Illinois; University of Chicago Medicine, Chicago, Illinois; University of Chicago, Chicago, Illinois; University of Chicago, Chicago, Illinois; University of Chicago Medicine, Chicago, Illinois; University of Chicago Medicine, Chicago, Illinois

## Abstract

**Background:**

Rates of congenital syphilis (CS) in the United States are rising sharply. As research has identified inadequate maternal syphilis treatment as a key cause, we aimed to investigate factors underlying this gap through retrospective patient data and interviews with prenatal providers.

Congenital syphilis (CS) diagnosis classification of infants delivered at Chicago-area tertiary care center from 2011-2023, grouped by maternal syphilis penicillin treatment course

**Figure:**
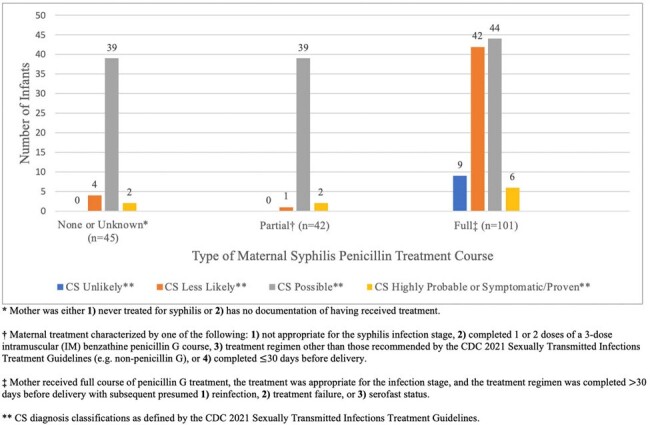

**Methods:**

We conducted a retrospective electronic health record chart review of mothers with syphilis during pregnancy and their infants treated for CS at a large Chicago tertiary care center from 2011-2023 to describe demographic variables, Centers for Disease Control and Prevention CS diagnosis classification, and infant and maternal treatment details. Bivariate analyses were used to compare patient predictors and maternal syphilis treatment. We also recruited a subset of the sample’s prenatal providers to participate in semi-structured interviews about their perspectives on the rise of CS. Qualitative themes were identified using a content analysis approach.

Type of syphilis treatment received by mothers of infants treated for CS at a Chicago-area tertiary care center from 2011-2023, grouped by location of prenatal care

**Figure:**
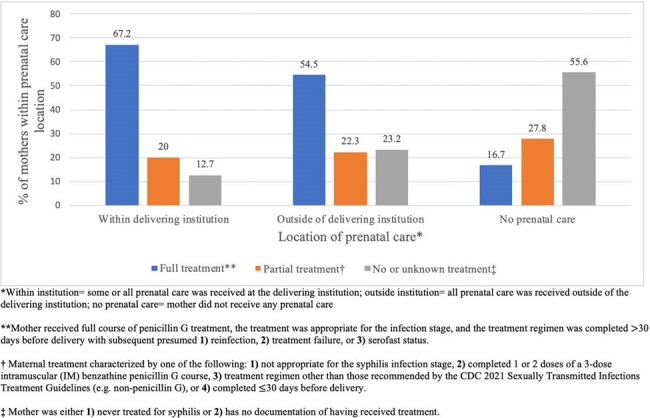

**Results:**

188 infants were identified. 45 (24%) pregnancies received no or unknown penicillin treatment prior to delivery, 42 (22%) partial treatment, and 101 (54%) full treatment. 9 (5%) infants were classified as unlikely CS, 47 (25%) as less likely, 122 (65%) as possible, and 10 (5%) as highly probable/proven. There were no significant differences between covariates based on maternal treatment, except for prenatal care (full treatment represented 67.3% of mothers who received care within the delivering institution vs. 54.5% outside vs. 14.3% none, p< 0.01) and CS type (86.7% of infants with possible CS had no maternal treatment vs. 92.9% partial vs. 43.6% full, p< 0.01). 10 providers were interviewed, most of whom observed inadequate treatment. Interviews revealed multiple social factors, including transportation, childcare, and appointment burden, as contributing to incomplete treatment courses. Communication difficulties, health literacy, CS-related stigma, and internal logistical challenges were further cited.

Demographics of mothers with syphilis during their pregnancies and their infants treated for congenital syphilis (CS) at a large Chicago tertiary care center from 2011-2023

**Figure:**
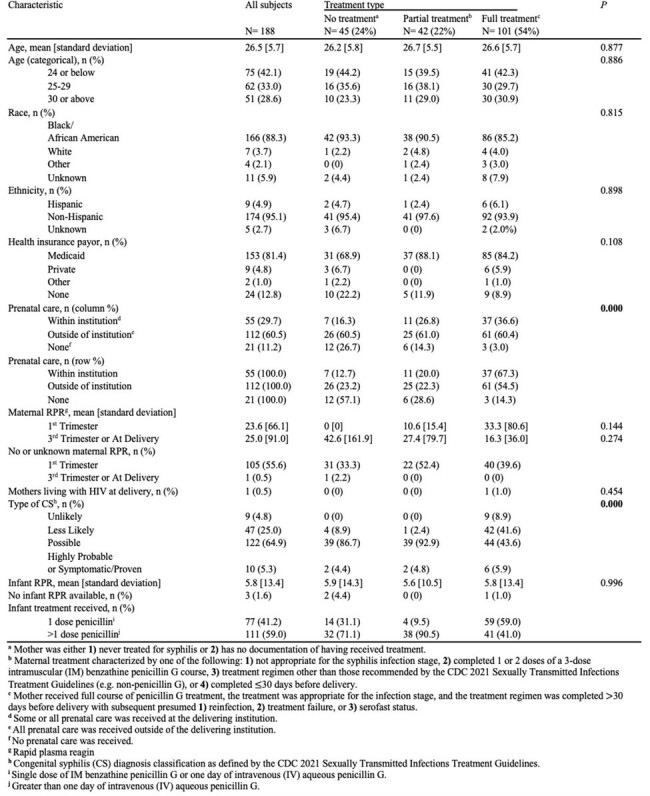

**Conclusion:**

Mothers with prenatal care had a significantly higher proportion receive full treatment compared to those with no care. Transportation, appointment burden, health literacy, stigma, and patient-provider communication were all identified by providers as contributing to the treatment gap and rise of CS.

**Disclosures:**

**Jessica Ridgway, MD**, Gilead Sciences: Expert Testimony

